# A universal medium bridging the shake-flask to fermenter gap in *Pichia pastoris* for enhanced zearalenone lactonase production

**DOI:** 10.3389/fmicb.2026.1854154

**Published:** 2026-07-01

**Authors:** Hujun Liu, Wen Du, Xiaojiao Chang, Yifan Zhao, Wenfu Wu, Changpo Sun

**Affiliations:** 1 College of Biological and Agricultural Engineering, Jilin University, Changchun, Jilin, China; 2 Academy of National Food and Strategic Reserves Administration, Beijing, China

**Keywords:** corn steep powder, medium optimization, *Pichia pastoris*, shake-flask to fermenter, transcriptomics, zearalenone lactonase

## Abstract

**Introduction:**

Zearalenone (ZEN) lactonase is a commercially promising enzyme that catalyzes the conversion of zearalenone into less toxic metabolites and has been expressed in *Pichia pastoris*. However, differences in culture media between shake-flask and fermenter systems hinder efficient production.

**Methods:**

Using a data-driven approach combined with rational analysis, an optimized medium (FM4CSP) was developed for both systems.

**Results:**

ZEN lactonase activity reached 25.87 ± 0.52 U/mL in shake flasks with FM4CSP medium, whereas it was negligible in the conventional FM22 medium. To elucidate the underlying mechanisms, comparative transcriptomic and nitrogen composition analyses were conducted. The results revealed that organic nitrogen sources enhance heterologous protein expression by alleviating energy metabolic stress under oxygen-limited conditions. Large peptides serve as core active components, acting as slow-release nitrogen sources that maintain stable amino acid availability. The balanced peptide profile in complex nitrogen sources triggered metabolic reprogramming, including downregulation of reducing equivalent-generating pathways to prevent NADH accumulation and upregulation of oxidative phosphorylation to match energy supply with oxygen availability. The effectiveness of FM4CSP was further validated in a 30 L fermenter, where ZEN lactonase activity reached 327.56 ± 1.78 U/mL, representing a 1.59-fold increase compared with the conventional FM22 medium.

**Conclusion:**

This study developed a novel universal medium (FM4CSP) for both shake-flask and fermenter systems using a cost-effective corn steep powder (CSP) as a slow-release nitrogen source. This medium effectively bridges the compatibility gap between both systems and provides a scalable strategy for heterologous protein production in *P. pastoris*.

## 1 Introduction

Zearalenone (ZEN), a secondary metabolite produced by toxigenic fungi such as *Fusarium graminearum, Fusarium culmorum, and Fusarium cerealis*, has a chemical structure that closely resembles that of mammalian estrogen (Ropejko and Twarużek, 2021). By competitively binding to estrogen receptors, ZEN induces reproductive dysfunction and organ damage, including abortion in females and reduced sperm viability in males (Lv et al., 2025). Given its high toxicity and widespread occurrence, effective detoxification strategies are urgently needed. Zearalenone lactonase (ZEN lactonase) has attracted considerable attention because it specifically catalyzes the ring opening of ZEN, yielding low-toxicity metabolites (Song et al., 2025). Despite successful expression of ZEN lactonase in heterologous systems such as *Escherichia coli* (Fu et al., 2021), *Lactobacillus reuteri* (Yang et al., 2017), and *Pichia pastoris* (Xiang et al., 2016), low expression levels have severely limited its industrial application.

The *P. pastoris* expression system, especially the Mut^+^ strain that utilizes the strong P*_*AOX1*_* promoter (in contrast to Mut^+^ strains that rely on the weaker P*_*AOX2*_*), is characterized by efficient secretory expression and well-established industrial processes, making it a preferred platform for recombinant protein production (Barone et al., 2023). However, its performance is constrained by significant differences between shake-flask and industrial culture media formulations, an issue that has persisted for decades. In shake-flask cultivation, complex media such as BMGY and BMMY, which are rich in organic nitrogen sources (e.g., yeast extract and peptone), are commonly employed to maximize recombinant protein yield. In contrast, fermenter systems predominantly use mineral salts (MS) media (e.g., FM22, D’Anjou medium, and BSM), in which organic nitrogen sources are replaced with inorganic ammonium salts to reduce costs (Bolmanis et al., 2023). Compared with BMMY medium, MS media differ in three key aspects. First, the higher inorganic salt concentration increases osmotic pressure and promotes precipitation during sterilization, thereby compromising physicochemical stability (Ghosalkar et al., 2008; Zhu et al., 2021). Second, MS media lack organic nitrogen sources, which are essential for microbial growth and heterologous protein expression, as demonstrated in previous studies (Velastegui et al., 2019; Zhu et al., 2021). Third, the absence of a buffering system results in substantial pH fluctuations during shake-flask cultivation. These limitations render MS media unsuitable for direct application in shake-flask systems, which lack pH control and typically exhibit low dissolved oxygen (DO) levels. Consistent with these observations, our preliminary experiments confirmed a significant discrepancy between shake-flask and fermenter cultures: when FM22 medium was used, ZEN lactonase activity exceeded 200 U/mL in the fermenter, whereas negligible enzyme induction and production were observed in shake flasks. This highlights the absence of a universal medium compatible with shake-flask and fermenter cultivation.

Several studies have attempted to address this challenge. For example, using low-inorganic-salt formulations or reducing inorganic salt concentrations in MS media can mitigate these limitations and improve heterologous protein expression (Matthews et al., 2018; Zhu et al., 2021). Similarly, incorporating organic nitrogen sources into inorganic salt-based media enhances microbial growth and heterologous protein production in shake flasks (Velastegui et al., 2019; Kaushik et al., 2020; Žitkus et al., 2025). However, current culture systems exhibit poor compatibility between laboratory shake-flask and industrial fermenter conditions, which limits the reliable evaluation and scale-up of recombinant protein production. Furthermore, the mechanisms underlying the differential performance of MS media across these systems remain unclear. To address these challenges, we hypothesized that systematic medium optimization, guided by comparative analysis with BMGY medium and supported by transcriptomic analysis, may elucidate expression differences between the two systems and enable the development of a universal culture medium.

Therefore, we aimed to investigate medium optimization for ZEN lactonase production in both shake-flask and fermenter systems, with key components identified through design of experiments (DoE). In addition, transcriptomic analyses were conducted to elucidate the mechanisms underlying culture-dependent differences in expression.

## 2 Materials and methods

### 2.1 Strains, media, and shake-flask cultivation

Recombinant *P. pastoris* GS115/Mut^+^/ZHD101, which expresses ZEN lactonase under the control of P*_*AOX1*_*, was stored in 20% (v/v) glycerol at –80°C in our laboratory.

For shake-flask cultivation, a single colony was inoculated into 50 mL of YPD medium (1% yeast extract, 2% peptone, and 2% glucose) in 250 mL flasks and incubated at 30°C with shaking at 200 rpm for 24 h. The resulting seed culture was used to inoculate 50 mL of enrichment medium at 5% (v/v), and the mixture was incubated for 16 h. Cells were harvested by centrifugation at 10,000 × *g* for 10 min at 4°C. The supernatant was aseptically discarded, and the cell pellet was resuspended in 50 mL of induction medium. Cultures were then incubated at 30°C with shaking at 200 rpm for 96 h, with methanol supplemented to a final concentration of 10 g/L every 24 h to maintain induction.

The BMGY enrichment medium was prepared as previously described, and the BMMY induction medium was obtained by replacing glycerol with methanol (Li C. et al., 2024). FM22 medium was used as previously reported (Bolmanis et al., 2023). In this study, the concentrations of inorganic salts in FM22 medium were reduced four-fold, except for (NH_4_)_2_SO_4_, to generate FM224 medium, thereby reducing precipitation and osmotic stress (Zhu et al., 2021). The FMG224 enrichment medium contained KH_2_PO_4_ (10.7 g/L), (NH_4_)_2_SO_4_ (5.0 g/L), K_2_SO_4_ (3.6 g/L), MgSO_4_⋅7H_2_O (2.9 g/L), CaSO_4_⋅2H_2_O (0.25 g/L), PTM_5_ (0.5 mL/L), biotin (1 × 10^–4^ g/L), and glycerol (10.0 g/L). The FMM224 induction medium was prepared by replacing glycerol in FMG224 with methanol (10 g/L). The pH of the enrichment and induction media was adjusted to 6.0 and 6.5, respectively, using 5 M NaOH. The PTM5 trace element solution contained FeSO_4_⋅7H_2_O (22.0 g/L), ZnCl_2_ (7.0 g/L), MnSO_4_⋅H_2_O (3.0 g/L), CuSO_4_⋅5H_2_O (2.0 g/L), CaSO_4_⋅2H_2_O (0.5 g/L), CoCl_2_ (0.5 g/L), Na_2_MoO_4_⋅2H_2_O (0.2 g/L), KI (0.089 g/L), H_3_BO_3_ (0.02 g/L), and H_2_SO_4_ (1 mL/L) and was stored at 4°C.

### 2.2 Medium optimization

#### 2.2.1 Plackett-Burman design

To identify critical variables affecting ZEN lactonase production, a Plackett-Burman design (PBD) was employed to screen and evaluate medium constituents. Nine factors were assessed at two levels: yeast extract (0 and 1.00 g/L), tryptone (0 and 2.00 g/L), CaSO_4_⋅2H_2_O (0.10 and 0.25 g/L), (NH_4_)_2_SO_4_ (5.00 and 6.25 g/L), MgSO_4_⋅7H_2_O (2.90 and 3.63 g/L), PTM_5_ (0.33 and 0.5 mL/L), biotin (0.10 and 0.125 mg/L), KH_2_PO_4_ (10.70 and 13.40 g/L), and K_2_SO_4_ (3.60 and 4.50 g/L).

#### 2.2.2 Single-factor experiments

Based on the PBD results, tryptone (T) was replaced (at equivalent nitrogen content) with various nitrogen sources, including high-percentage amino acids in tryptone [HTA; e.g., arginine (Arg), phenylalanine, glutamic acid, leucine (Leu), aspartic acid, and proline (Pro)], high-percentage amino acids in the target protein (HPA; e.g., serine, Pro, glycine, threonine, Leu, alanine, and valine), and amino acids with high synthetic energy [HEA; e.g., cysteine (Cys), tryptophan, phenylalanine, tyrosine (Tyr), histidine (His), and methionine (Met)] (Heyland et al., 2011). Additional nitrogen sources included glutamine and complex nitrogen sources such as peptone (P), soy peptone (SP), fish peptone (FP), beef peptone (BP), casein hydrolysate (CH), and CSP.

The concentrations of methanol, CSP, and (NH_4_)_2_SO_4_ were further varied to optimize ZEN lactonase production. Methanol content was tested at 5, 10, 15, 20, 30, and 40 g/L; CSP content at 4, 8, 12, 16, 20, 24, and 28 g/L; and (NH_4_)_2_SO_4_ content at 3.0, 3.5, 4.0, 4.5, and 5.0 g/L. Additionally, because initial cell density influences nutrient utilization and has been reported to significantly affect heterologous protein expression in *P. pastoris* (Jia et al., 2017b), the initial cell density was evaluated at OD_600_ values of 5, 10, 20, 30, and 40. Each single-factor experiment was conducted independently, with all other variables held constant.

#### 2.2.3 Central composite design-response surface methodology

Based on the results of the PBD and single-factor experiments, a central composite design-response surface methodology (CCD-RSM) was applied to optimize CSP concentration, methanol concentration, and initial cell density (OD_600_) (Nuge et al., 2023). The region of optimal ZEN lactonase activity was first approached using the steepest ascent method. Subsequently, CCD-RSM was performed using five coded levels (–1.682, -1, 0, 1, and 1.682) for each variable. The design comprised 20 experimental runs, including six center-point replicates (Table 1). Design-Expert 13 (Stat-Ease Inc.) was used to generate the experimental design and construct a regression model to predict optimal variable combinations for ZEN lactonase production. Model adequacy was evaluated using an analysis of variance (ANOVA) and a lack-of-fit test. The CCD-RSM model was validated through a confirmatory experiment.

**TABLE 1:** Factor levels for CCD-RSM.

Factor	Level				
	–1.682	–1	0	1	1.682
A-CSP (g/L)	14.60	16.00	18.00	20.00	21.40
B-Initial cell density	26.60	30.00	35.00	40.00	43.40
C-Methanol (g/L)	11.60	15.00	20.00	25.00	28.40

### 2.3 Mechanism of organic nitrogen source impact: a systematic investigation

#### 2.3.1 Peptide composition of organic nitrogen sources and their effects on ZEN lactonase production

To investigate the effects of different organic nitrogen sources on ZEN lactonase production, CSP in the optimized medium (FM4CSP) was individually replaced with tryptone and yeast extract at equivalent total nitrogen content, yielding modified media designated FM4T and FM4YE, respectively. A nitrogen-free basal medium (FM4), prepared by omitting CSP from FM4CSP, served as the control.

To elucidate the underlying mechanisms, the peptide molecular weight distributions of yeast extract, tryptone, and CSP were analyzed. Tryptone was further fractionated into small-molecule peptides (SMPs; < 5 kDa) and large-molecule peptides (LMPs; > 5 kDa) using a 5 kDa ultrafiltration membrane (Fontana et al., 2025). The resulting SMP and LMP fractions were directly used to replace tryptone in FM4T medium, generating FM4SMP and FM4LMP media, respectively. The total organic nitrogen content in each medium was determined using the Dumas combustion method.

#### 2.3.2 Mechanistic analysis at the transcriptome level

Comparative transcriptomic analyses were conducted to investigate metabolic pathways associated with ZEN lactonase production in cells cultured in FM4, FM4YE, FM4T, and FM4CSP. Transcriptional profiles were comprehensively analyzed, with pairwise comparisons performed between the following groups: FM4 versus FM4YE (MY), FM4 versus FM4T (MT), FM4 versus FM4CSP (MC), FM4YE versus FM4CSP (YC), and FM4YE versus FM4T (YT). Cells from each condition were collected in three biological replicates, immediately snap-frozen in liquid nitrogen, and stored at –80°C for subsequent RNA isolation and sequencing.

Total RNA isolation, library construction, and high-throughput sequencing were performed by Beijing Biomarker Technologies Co., Ltd. The BMKCloud platform^1^ was used for bioinformatic analysis of the sequencing data. The genomic DNA of *Komagataella phaffii* GS115 was used as the reference genome. Transcript expression levels were quantified as fragments per kilobase of transcript per million mapped reads. Differentially expressed genes (DEGs) were identified using DESeq2 and EBSeq, with thresholds of | log_2_ fold change| ≥ 1.5 and *p* < 0.05. The KEGG database was used for functional annotation and metabolic pathway enrichment analysis of the identified DEGs.

### 2.4 Scale-up verification in a 30 L fermenter

To validate the industrial applicability of the optimized culture medium, fed-batch cultivation was performed in a 30 L fermenter with an initial working volume of 10 L. The temperature was maintained at 30°C throughout the process. The pH was controlled at 6.0 during the growth phase and 6.5 during the methanol induction phase through the automatic addition of 25% ammonium hydroxide. FMG224 was employed as the seed medium, with an inoculation rate of 10% (v/v). The cultivation process comprised three phases: batch growth (B), fed-batch growth (FB), and methanol induction (M0–M4). During the batch growth phase, cells were cultured until complete glycerol depletion at approximately 16 h, as indicated by a sharp increase in DO. During the FB growth phase, 2 L of 50% (w/w) glycerol supplemented with PTM_5_ (4 mL/L) and biotin (4.0 × 10^–4^ g/L) was fed using a DO-stat strategy to maintain DO at 20%. For the methanol induction phase, cells were first adapted by feeding methanol (100%) supplemented with PTM_5_ (4 mL/L) and biotin (4.0 × 10^–4^ g/L) to a concentration of 2 g/L. This was followed by the induction phase, during which methanol concentration was maintained at 10 g/L using an online methanol sensor. Samples were collected at key time points: initiation (B0) and completion (B1) of the batch phase, completion of the FB, initiation of methanol induction (M0), and at approximately 24, 48, 72, and 96 h during methanol induction (M1–M4).

### 2.5 Analytical methods

Biomass was monitored by measuring absorbance at 600 nm (A_600_) (Zhou et al., 2021), and wet cell weight was determined as described by Li H. et al. (2024).

Culture supernatants were collected by centrifugation at 10,000 × *g* for 5 min at 4°C. Extracellular ZEN lactonase was analyzed by SDS-PAGE (Burgard et al., 2017). For the activity assay, crude enzyme was prepared by diluting the supernatant with PBS (pH 7.4). Reaction mixtures containing 10 μg ZEN, 390 μL PBS, and 100 μL crude enzyme were incubated at 37°C for 10 min. Heat-inactivated enzyme served as the negative control. Reactions were terminated by adding 500 μL acetonitrile, and residual ZEN was quantified by HPLC (Waters) using a C18 column (250 × 4.6 mm, 5 μm) with acetonitrile: water (50:50, v/v) as the mobile phase at a flow rate of 1.0 mL/min. Fluorescence detection was performed at *λ_*ex*_* = 274 nm and *λ_*em*_* = 440 nm at 35°C.

One unit of ZEN lactonase activity was defined as the amount of enzyme required to degrade 1 μg ZEN per min under standard conditions. Activity was calculated as follows (Equation 1):


ZEN lactonase activity(U/mL)=(M−0M)1×N/(T×V) (1)

where *M*0 is the amount of ZEN in the negative control (μg), *M*1 is the residual ZEN (μg), *N* is the dilution factor, *T* is the reaction time (min), and *V* is the volume of crude enzyme (mL).

The specific growth rate (μ) and specific production rate (q_p_) were calculated as described previously (Changming et al., 2022).

The amino acid profiles of the organic nitrogen sources were determined using an automatic amino acid analyzer (Hitachi, LA8080, Japan) following acid hydrolysis with 6 M HCl at 110°C for 22 h, as previously described by Lin et al. (2018).

Total nitrogen content was determined using the Dumas combustion method with an elemental rapid nitrogen analyzer (Elementar, Rapid N exceed). Briefly, 2 mL of the sample solution was mixed with soluble starch and dried to constant weight at 40°C. Aspartic acid (10.52% N) was used as the calibration standard. Combustion parameters included a furnace temperature of 950°C, an oxygen pressure of 0.25 MPa, and a CO_2_ carrier gas pressure of 0.14 MPa (Vershinina et al., 2025).

The peptide molecular weight distribution was analyzed by gel permeation chromatography (GPC) using an Agilent 1260 Infinity II system equipped with a refractive index detector and a multi-angle light scattering detector. Samples (1–3 mg/mL in the mobile phase) were separated on a PL aquagel-OH Mixed-H column (7.5 × 300 mm, 8 μm) using 0.1 M sodium nitrate containing 0.01% (w/v) NaN_3_ as the mobile phase at a flow rate of 1 mL/min and 45°C. Molecular weights were calculated using Agilent GPC/SEC software with pullulan standards (Lin et al., 2019).

### 2.6 Statistical analysis

All shake-flask experiments were performed in triplicate; fermenter data are from single runs with technical replicates. Data were analyzed using Excel 2019 and Design-Expert 13.0 and plotted using Origin 2022. Results are presented as mean ± standard deviation.

## 3 Results

### 3.1 Optimization of a universal medium for shake-flask and fermenter systems

#### 3.1.1 Identification of key limiting factors affecting ZEN lactonase expression in shake flasks

To address the common challenge of inconsistent media performance between shake-flask and fermenter systems, the fermenter-oriented FM22 medium was modified to FM224 by four-fold dilution of most inorganic salts to reduce precipitation and osmotic stress. In this study, FMG224 was used as the enrichment medium, and FMM224 served as the basal induction medium for subsequent optimization.

Preliminary experiments revealed that basal FMM224 yielded no detectable ZEN lactonase activity, whereas BMMY supported robust expression. Even after stabilizing the pH of FMM224 through periodic NaOH adjustment (6.2–6.5), ZEN lactonase activity reached only 1.54 ± 0.06 U/mL, which is substantially lower than the 12.58 ± 1.00 U/mL observed in BMMY (Supplementary Table 1). These findings indicate that FMM224 lacks the key components required for efficient ZEN lactonase production.

To identify the limiting factors, a Plackett-Burman design was applied to screen critical medium components affecting ZEN lactonase production. The experimental data (Supplementary Table 2) were analyzed using Design-Expert 13.0, and the results are summarized in Table 2. Among the nine components evaluated, only tryptone (B) had a statistically significant effect on ZEN lactonase production (*p* < 0.05), indicating its essential role in enhancing enzyme production. In contrast, PTM5 trace elements and biotin did not significantly affect ZEN lactonase expression, suggesting that they can be omitted to simplify medium preparation and reduce costs. Based on the PBD results, tryptone [2% (w/v)] was added to FMM224 to formulate FMM224T, which was used in subsequent optimization experiments.

**TABLE 2:** Statistical analysis of the Plackett-Burman design.

Source	Sum of squares	*df*	Mean square	*F*-value	*P*-value
Model	80.72	9	8.97	49.53	0.0199
A-yeast extract	3.04	1	3.04	16.79	0.0547
B-tryptone	74.60	1	74.60	411.97	0.0024
C-CaSO_4_	0.1728	1	0.1728	0.9543	0.4317
D-(NH_4_)_2_SO_4_	1.86	1	1.86	10.25	0.0853
E-MgSO_4_	0.1875	1	0.1875	1.04	0.4159
F-PTM_5_	0.0705	1	0.0705	0.3895	0.5963
G-biotin	0.3960	1	0.3960	2.19	0.2773
H-KH_2_PO_4_	0.2187	1	0.2187	1.21	0.3864
I-K_2_SO_4_	0.1776	1	0.1776	0.9809	0.4264

#### 3.1.2 Single-factor optimization of the induction medium

**FIGURE 1:**
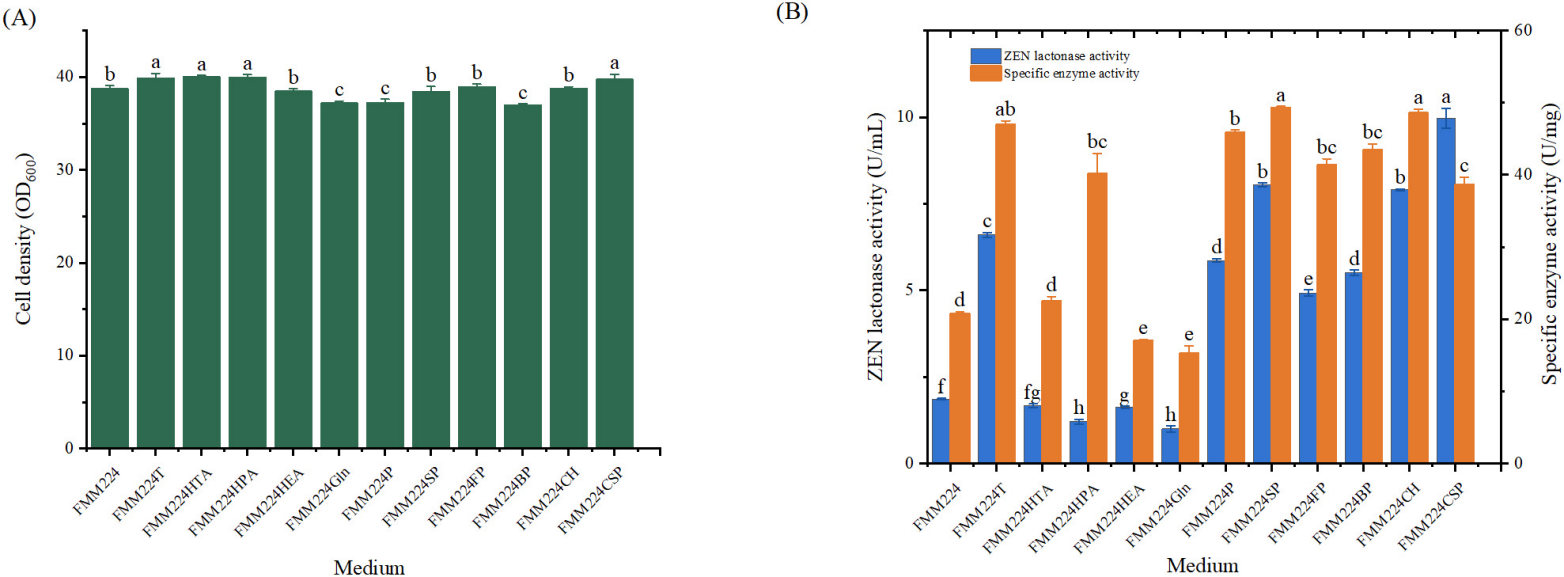
Influence of nitrogen sources on cell density **(A)** and ZEN lactonase production **(B)**. Complex nitrogen sources, especially CSP, soybean peptone (SP), and casein hydrolysate (CH), significantly enhanced ZEN lactonase activity compared to amino acid mixtures or the control. Different letters indicate statistically significant differences between groups (*p* < 0.05).

The PBD results indicated that organic nitrogen sources, especially tryptone, play a decisive role in ZEN lactonase expression. Previous studies have demonstrated that *P. pastoris* preferentially utilizes amino acids from organic nitrogen sources, followed by ammonium nitrogen (Wang et al., 2005). Notably, different organic nitrogen sources—and even the same source derived from different origins—exhibit considerable variability in their effects on heterologous protein expression (Mao et al., 2015; Lee, 2018). To identify cost-effective alternatives, tryptone in FMM224T was replaced with various nitrogen sources as described in the Materials and Method, and the results are presented in Figure 1.

As shown in Figure 1A, the nitrogen source type had a limited effect on cell growth, with OD_600_ values ranging from 37.05 ± 0.10 to 40.07 ± 0.14. P, HTA, HPA, and CSP significantly increased biomass (*p* < 0.05). In contrast, none of the amino acid mixtures improved ZEN lactonase expression (Figure 1B). Complex nitrogen sources, especially CSP, CH, and SP, significantly enhanced enzyme activity, reaching 9.96 ± 0.03, 7.90 ± 0.02, and 8.04 ± 0.06 U/mL, respectively. These values were markedly higher than those of FMM224T (6.60 ± 0.07 U/mL, *p* < 0.05). Specific enzyme activity was also higher in cultures supplemented with complex nitrogen sources than in those containing amino acid mixtures, with the highest value observed in the SP group (49.30 ± 0.22 U/mg). The relatively high specific activity in the HPA group (40.49 ± 1.61 U/mg) suggests that amino acids abundant in the target protein may favor its expression.

**FIGURE 2:**
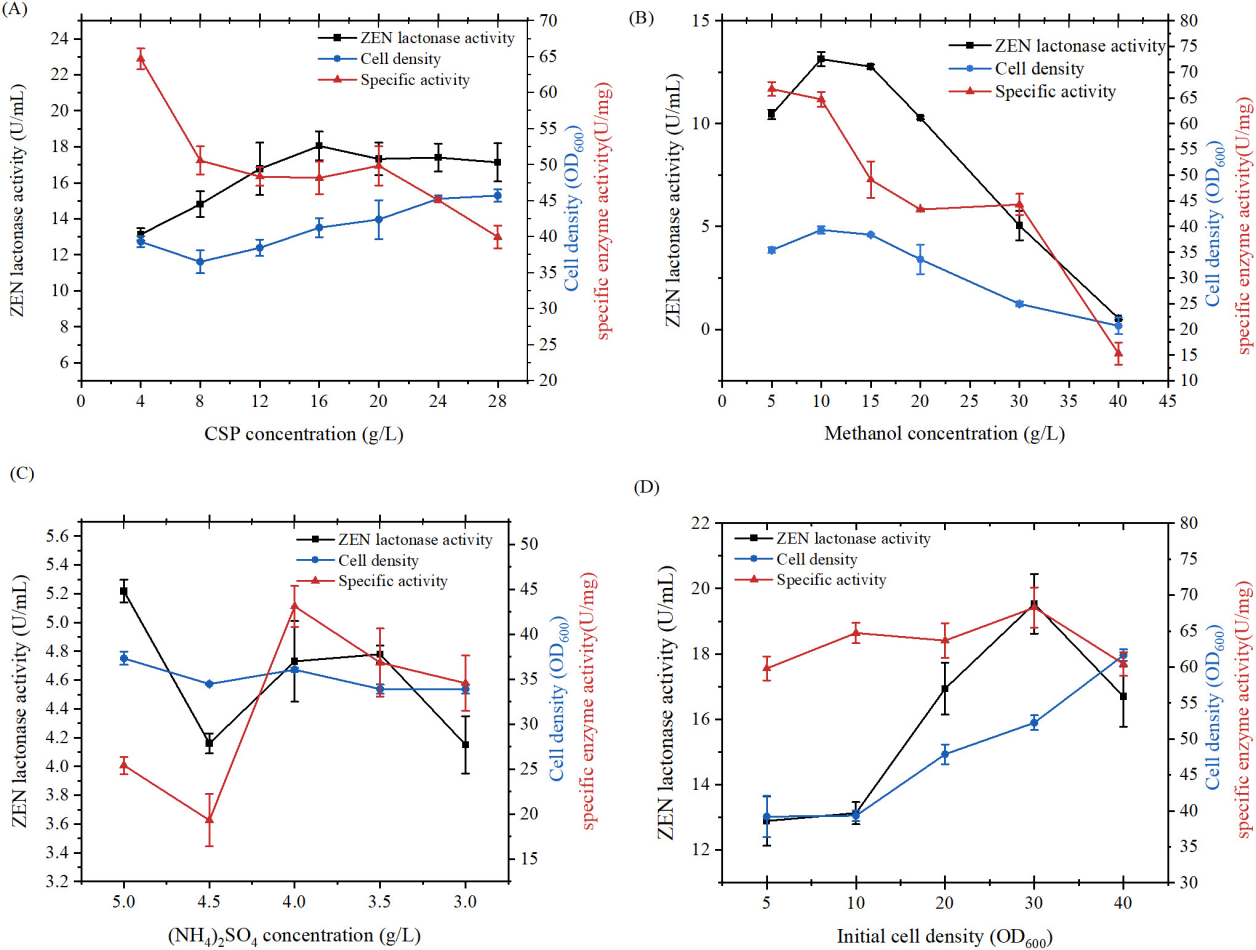
Single-factor optimization of **(A)** CSP concentration, **(B)** methanol concentration, **(C)** (NH_4_)_2_SO_4_ concentration, and **(D)** initial cell density. The optimal CSP concentration was 16 g/L, methanol concentration 10 g/L, (NH*4*)*2*SO*4* concentration 5 g/L, and initial cell density OD_600_ = 30 for maximum enzyme activity.

The concentrations of methanol, CSP, and (NH_4_)_2_SO_4_, as well as the initial cell density, were subsequently optimized. As shown in Figure 2A, ZEN lactonase activity and cell density increased with increasing CSP concentration, with ZEN lactonase activity reaching peak values of 18.05 ± 0.80 U/mL at 16 g/L and cell density reaching 45.72 ± 0.86 OD_600_ at 28 g/L. For methanol (Figure 2B), both parameters reached maximum values at 10 g/L (39.33 ± 0.74 OD_600_ and 13.13 ± 0.34 U/mL). Increasing the methanol concentration to 15 g/L resulted in declines in both cell density and ZEN lactonase activity, followed by a rapid decrease at higher concentrations. These results suggest that methanol exerts toxic effects on cells at concentrations above 15 g/L, thereby inhibiting cell growth and enzyme production. (NH_4_)_2_SO_4_ exhibited an overall negative effect (Figure 2C), with the highest ZEN lactonase activity observed at 5 g/L, which was selected for subsequent experiments. Regarding initial cell density (Figure 2D), both ZEN lactonase activity and final biomass increased with increasing initial cell density, with enzyme activity peaking at 19.53 ± 0.91 U/mL at an initial OD_600_ of 30, while biomass continued to increase to 61.64 ± 0.80 OD_600_. Specific enzyme activity was significantly decreased at high concentrations of CSP and methanol but increased at elevated (NH_4_)_2_SO_4_ concentrations. In contrast, initial cell density had a minimal effect on specific activity.

#### 3.1.3 CCD model fitting and response surface analysis

Based on the single-factor results, CSP concentration, methanol concentration, and initialcell density were selected for optimization using the steepest ascent method. As shown in Table 3, the optimal region was approached near the fourth experimental run. Accordingly, the factor levels of this run were selected as the central point for the CCD-RSM design.

**TABLE 3:** Design and results of the steepest ascent method.

Exp. S/N	CSP concentration (g/L)	Initial cell density (OD_600_)	Methanol concentration (g/L)	ZEN lactonase activity (U/mL)
1	12	20	5	19.07 ± 0.32
2	14	25	10	23.84 ± 0.15
3	16	30	15	24.03 ± 0.28
4	18	35	20	25.73 ± 0.10
5	20	40	25	21.32 ± 0.41

A total of 20 experimental runs were generated using CCD-RSM. To balance experimental efficiency and statistical reliability, factorial and axial points were not replicated, whereas the center point was repeated six times to estimate pure experimental error. A quadratic multivariate regression model was fitted to the data in Table 4 using Design-Expert 13.0. Based on these results, a second-order polynomial model for ZEN lactonase activity was obtained (Equation 2).


Y=25.58+0.76⁢A+0.69⁢B−1.83⁢C+0.27⁢A⁢B−0.32⁢A⁢C (2)

+1.28BC−0.81A−21.50B−21.59C2

**TABLE 4:** Experimental design and results of the central composite design.

Run	CSP (g/L)	Initial cell density (OD_600_)	Methanol (g/L)	ZEN lactonase activity (U/mL)
1	18.00	35.00	20.00	25.11
2	20.00	40.00	25.00	22.04
3	16.00	30.00	15.00	23.48
4	20.00	30.00	15.00	24.44
5	18.00	35.00	28.41	18.86
6	14.64	35.00	20.00	22.20
7	16.00	40.00	15.00	21.63
8	21.36	35.00	20.00	24.79
9	18.00	26.59	20.00	20.99
10	18.00	35.00	20.00	25.17
11	16.00	40.00	25.00	21.24
12	20.00	30.00	25.00	17.67
13	18.00	35.00	11.59	23.73
14	18.00	35.00	20.00	26.03
15	18.00	35.00	20.00	26.22
16	18.00	43.41	20.00	22.10
17	18.00	35.00	20.00	25.71
18	20.00	40.00	15.00	24.95
19	18.00	35.00	20.00	25.17
20	16.00	30.00	25.00	16.72

Runs 1, 10, 14, 15, 17, and 19 represent the center-point replicates used to estimate pure error.

**FIGURE 3:**
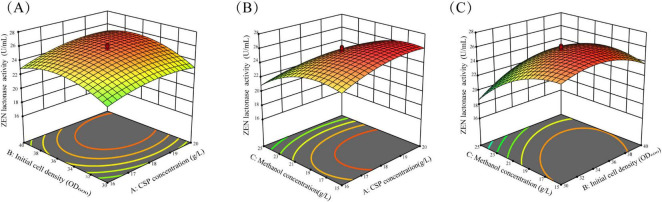
3D response surfaces of ZEN lactonase activity: **(A)** CSP concentration and initial cell density; **(B)** CSP concentration and methanol concentration; **(C)** initial cell density and methanol concentration. A significant interaction between initial cell density and methanol concentration was observed; increasing the initial cell density shifted the optimal methanol concentration upward **(C)**.

Here, *Y* represents the predicted ZEN lactonase activity (U/mL), and *A*, *B*, and *C* denote the coded variables for CSP concentration (g/L), initial cell density (OD_600_), and methanol concentration (g/L), respectively. As illustrated in Figure 3, quadratic polynomial regression was used to generate the response surface.

The CCD-RSM model was evaluated using ANOVA. As shown in Table 5, the model was highly significant, with an *F*-value of 30.75 and *p* < 0.0001, suggesting that it adequately describes the relationship between the independent variables and ZEN lactonase activity. The lack-of-fit term was not significant (*F* = 3.30, *p* = 0.1083 > 0.05), confirming that the model fits the experimental data without systematic deviation. The pure error (mean square = 0.24, *df* = 5), estimated from six center-point replicates, indicates good experimental reproducibility. The coefficient of determination (*R*^2^ = 0.9651) shows that 96.51% of the variation in ZEN lactonase activity is explained by CSP concentration (g/L), initial cell density (OD_600_), methanol concentration (g/L), and their interactions. The remaining 3.49% of the variability was not accounted for by the model.

**TABLE 5:** Analysis of variance for the central composite design-response surface model.

Source	Sum of squares	*df*	Mean square	*F*-value	*P*-value Prob > F
Model	141.57	9	15.73	30.75	< 0.0001**
A	7.88	1	7.88	15.41	0.0028**
B	6.48	1	6.48	12.67	0.0052**
C	45.78	1	45.78	89.48	< 0.0001**
AB	0.60	1	0.60	1.18	0.3029
AC	0.80	1	0.80	1.57	0.2385
BC	13.07	1	13.07	25.55	0.0005**
A2	9.55	1	9.55	18.67	0.0015**
B2	32.51	1	32.51	63.54	< 0.0001**
C2	36.45	1	36.45	71.25	< 0.0001**
Residual	5.12	10	0.51		
Lack-of-fit	3.93	5	0.79	3.30	0.1083
Pure error	1.19	5	0.24		

**P < 0.01 indicates highly significant effects in the ANOVA model.

In addition, CSP concentration, methanol concentration, and initial cell density each had a significant effect on ZEN lactonase production (*p* < 0.05). Among these factors, methanol concentration had the most significant effect (*p* < 0.0001), followed by CSP concentration (*p* = 0.0028) and initial cell density (*p* = 0.0052). A significant interaction between initial cell density and methanol concentration was observed (*p* = 0.0005; Table 5). Notably, increasing the initial cell density shifted the optimal methanol concentration upward (Figure 3C). A higher initial cell density reduces methanol availability per cell. Therefore, a higher absolute methanol concentration is required to achieve the optimal specific uptake rate, but the concentration must be kept within a safe range to avoid formaldehyde toxicity. Moreover, the response surface plots in Figure 3 exhibit pronounced curvature. Consistent with this observation, all quadratic terms in the model were statistically significant (*p* < 0.05; Table 5), and the coefficient of A^2^, B^2^ and C^2^ in (Equation 2) were negative. Collectively, these findings confirm the nonlinear relationship between the response variable and the independent factors. Based on the CCD-RSM analysis, the maximum predicted ZEN lactonase activity was 26.39 U/mL under the following optimal conditions: CSP concentration of 19.18 g/L, initial cell density of 35.08 (OD_600_), and methanol concentration of 16.86 g/L. To verify the accuracy of the response surface model, triplicate experiments were conducted under the predicted optimal conditions. The remaining medium components were fixed based on the single-factor results as follows: KH_2_PO_4_ (13.4 g/L), (NH_4_)_2_SO_4_ (5.0 g/L), CaSO_4_⋅2H_2_O (0.10 g/L), K_2_SO_4_ (3.6 g/L), MgSO_4_⋅7H_2_O (2.9 g/L), PTM5 (0.33 mL/L), and biotin (0.10 mg/L). This formulation was designated FM4CSP. The experimentally measured ZEN lactonase activity was 25.87 ± 0.52 U/mL, which closely agrees with the predicted value of 26.39 U/mL. These results confirm that the model is robust and suitable for optimizing ZEN lactonase production in *P. pastoris.*

### 3.2 Mechanistic insights into organic nitrogen source regulation of ZEN lactonase biosynthesis

Although organic nitrogen is essential for ZEN lactonase production in shake-flask cultures of *P. pastoris*, it is typically not required in fermenter systems. This discrepancy prompted further investigation into the regulatory mechanisms underlying nitrogen source-dependent ZEN lactonase production to enable rational medium design for fermenter applications. Three media containing different organic nitrogen sources were prepared as described in the Materials and Methods section. After 96 h of induction, ZEN lactonase activity in the organic nitrogen-free FM4 medium was only 2.78 ± 0.15 U/mL. In contrast, supplementation with organic nitrogen sources significantly enhanced ZEN lactonase expression (*p* < 0.05). Notably, CSP and tryptone yielded the highest ZEN lactonase activities, approximately ninefold greater than that observed in FM4 medium, whereas yeast extract resulted in a 5.3-fold increase relative to FM4 (Supplementary Figure 1).

#### 3.2.1 Analysis of organic nitrogen source composition and its effect on ZEN lactonase expression

To explore the underlying mechanism, the amounts of amino acids added to each medium were calculated, and their correlations with enzyme activity were analyzed (Supplementary Figure 2). ZEN lactonase activity was positively correlated with Arg (*r* = 0.6), Cys (*r* = 1.0), His (*r* = 1.0), Pro (*r* = 0.9), Met (*r* = 0.6), Leu (*r* = 0.5), and Tyr (*r* = 0.6). The *de novo* synthesis of amino acids requires varying energy inputs, typically quantified by the number of high-energy phosphate bonds consumed (Raiford et al., 2008). Among these seven amino acids, five are associated with high biosynthetic energy costs. However, the direct addition of high-energy-cost amino acids had a limited effect on ZEN lactonase activity under shake-flask conditions (Figure 1B).

**FIGURE 4:**
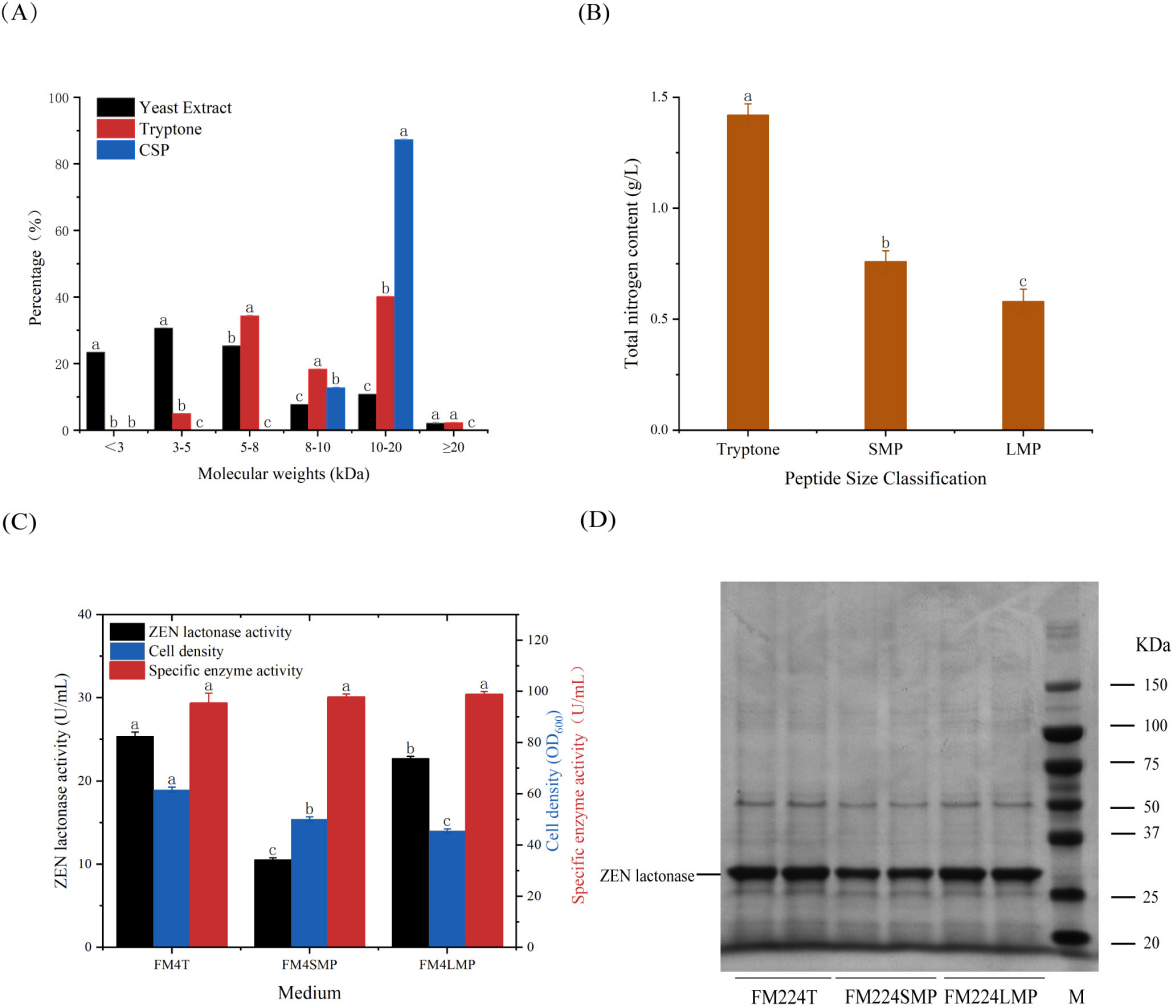
Peptide composition of organic nitrogen sources and their effects on ZEN lactonase production. **(A)** Peptide molecular weight distribution of the three organic nitrogen sources. **(B)** Total nitrogen content of tryptone and its fractionated components (SMP and LMP). **(C)** Effects of tryptone and its fractions on ZEN lactonase production and cell density. **(D)** SDS–PAGE analysis of ZEN lactonase production. Large peptides are beneficial for ZEN lactonase production, as demonstrated by the LMP fraction. Different letters indicate statistically significant differences between groups (*p* < 0.05).

Given the ability of *P. pastoris* to directly assimilate peptides, which may serve as more efficient nitrogen sources than free amino acids for protein production (Shang et al., 2017), we hypothesized that peptide molecular weight distribution plays a more critical role than amino acid composition or total nitrogen content. To test this hypothesis, the peptide distributions of the three organic nitrogen sources were analyzed (Figure 4A). The results indicated that yeast extract predominantly contained low-molecular-weight components ( < 8 kDa), accounting for 78.95% of the total, with peptides below 5 kDa comprising 54.04%. In contrast, CSP consisted almost entirely of high-molecular-weight components ( > 8 kDa, 100%; > 10 kDa, 87.28%). Tryptone exhibited a mixed distribution, with 4.96% of peptides below 5 kDa, 34.32% between 5 and 8 kDa, and 60.72% above 8 kDa (Figure 4A). Collectively, these findings indicate that a high proportion of high-molecular-weight peptides may be a key determinant of enhanced ZEN lactonase production.

To test the hypothesis that large peptides are the critical components, tryptone was fractionated using a 5 kDa ultrafiltration membrane to obtain SMP and LMP fractions (Fontana et al., 2025). These fractions were used to replace tryptone in FM4T medium, generating FM4SMP and FM4LMP media. The total organic nitrogen content was 1.42 ± 0.01 g/L in FM4T, 0.76 ± 0.01 g/L in FM4SMP, and 0.58 ± 0.01 g/L in FM4LMP (Figure 4B). As shown in Figure 4C, FM4SMP produced higher biomass than FM4LMP. However, the corresponding ZEN lactonase activity was significantly lower, reaching only 47% of that observed in FM4LMP. Notably, despite containing only 40.84% of the organic nitrogen present in FM4T, FM4LMP yielded a ZEN lactonase activity of 22.72 ± 0.24 U/mL, which is comparable to that of FM4T (25.37 ± 0.52 U/mL). No significant difference in specific ZEN lactonase activity was observed between FM4LMP and FM4T. These findings are consistent with the SDS-PAGE results in Figure 4D.

#### 3.2.2 Transcriptomic analysis

**FIGURE 5:**
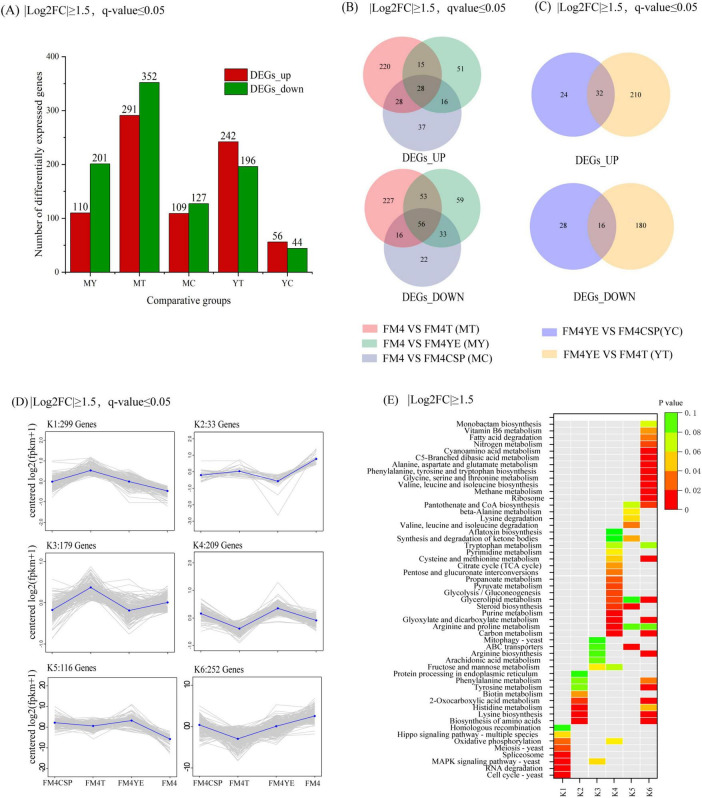
Differentially expressed genes (DEGs) profiles and co-expression analysis under different culture conditions. **(A)** Number of DEGs between media. **(B)** Venn diagram of DEGs comparing FM4 with organic nitrogen-supplemented media. **(C)** Venn diagram of DEGs in FM4Y versus FM4T and FM4Y versus FM4CSP. **(D)** Co-expression analysis showing clustering of DEGs into six clusters (K1–K6). **(E)** KEGG pathway enrichment of DEGs in each cluster. Organic nitrogen sources commonly downregulated energy-intensive amino acid biosynthesis, thereby alleviating metabolic burden under oxygen limitation.

Transcriptomic profiling revealed distinct expression patterns across the four media. Tryptone induced the most extensive transcriptional changes, with 643 DEGs identified relative to FM4, whereas yeast extract and CSP generated 311 and 236 DEGs, respectively (Figure 5A). Among the three organic nitrogen sources, 84 DEGs were commonly regulated and enriched in pathways related to GPI-anchor biosynthesis, steroid biosynthesis, and amino acid metabolism. An additional 48 DEGs were shared between the yeast extract and tryptone and were primarily associated with central carbon metabolism and amino acid metabolism (Figures 5B,C). To further characterize the transcriptional dynamics of DEGs across the four induction media, we performed co-expression analysis using k-means clustering with k = 6 (Figure 5D).

KEGG analysis revealed that, compared with FM4, supplementation with organic nitrogen sources consistently downregulated pathways related to amino acid biosynthesis as well as central carbon and energy metabolism (e.g., biosynthesis of amino acids, glycolysis/gluconeogenesis, fatty acid degradation, and methane metabolism) (clusters K2 and K6; Figures 5D,E). Additionally, it upregulated genes associated with cell cycle regulation, signal transduction, secretory pathways, and oxidative phosphorylation (clusters K1 and K5; Figures 5D,E). Specifically, K1 genes were mainly involved in pathways such as cell cycle-yeast, RNA degradation, MAPK signaling pathway—yeast, spliceosome, meiosis-yeast, oxidative phosphorylation, and hippo signaling pathway. However, K5 genes predominantly participated in steroid biosynthesis; ABC transporters; valine, leucine, and isoleucine degradation; and synthesis and degradation of ketone bodies. Notably, compared with other groups, supplementation of tryptone further suppressed metabolic pathways, including carbon metabolism, arginine and proline metabolism, glyoxylate and dicarboxylate metabolism, purine metabolism, steroid biosynthesis, and glycerolipid metabolism (cluster K4; Figures 5D,E).

**FIGURE 6:**
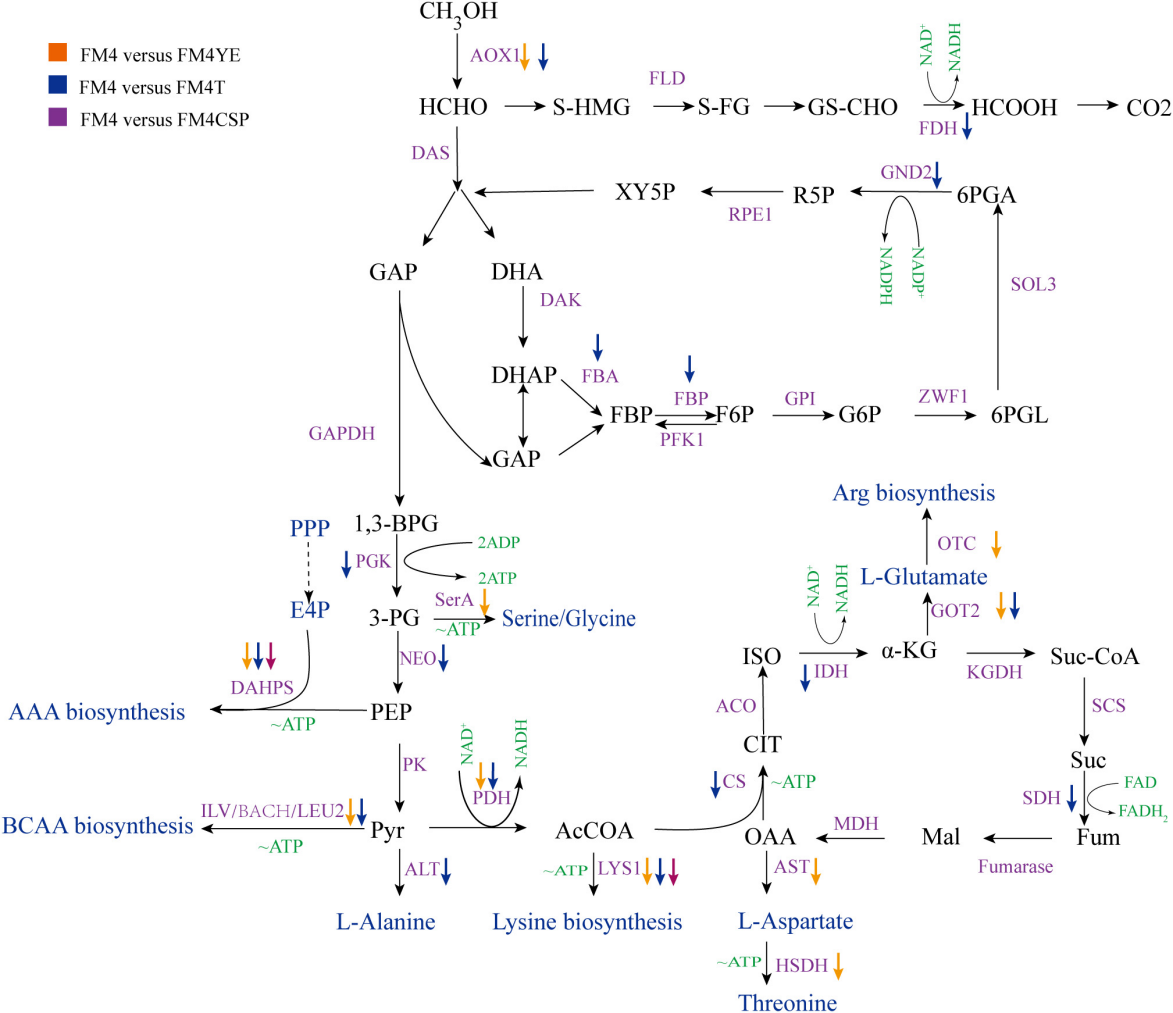
Schematic overview of key metabolic pathways and gene expression changes in *P. pastoris* under different organic nitrogen supplementation conditions (*q* ≤ 0.05). Yeast extract broadly downregulates amino acid biosynthesis and represses AOX1. CSP mainly downregulates high-energy-cost amino acid biosynthesis. Tryptone causes more extensive suppression, including pathways of methanol metabolism, central carbon metabolism, and reducing-equivalent generation.

We constructed a metabolic network to visualize the differences in metabolic regulation induced among groups with a specific organic nitrogen source. As shown in the metabolic network (Figure 6), the FM4YE group showed widespread downregulation of pathways related to amino acid biosynthesis. In addition, yeast extract supplementation downregulated the methanol oxidase gene (*AOX1*). In the FM4CSP group, downregulation was primarily limited to biosynthetic pathways for high-energy-cost aromatic amino acid (AAA) and lysine. In contrast, tryptone supplementation resulted in broader metabolic suppression, including amino acid biosynthesis pathways and key genes involved in methanol metabolism (*AOX1*, *FDH*), the pentose phosphate pathway (*FBA*, *FBP*, *GND2*), glycolysis (*PGK*, *NEO*, *PDH*), and the TCA cycle (*CS*, *IDH*, *SDH*). Notably, many of these downregulated genes (*FDH*, *GND2*, *PDH*, *IDH*, and *SDH*) are associated with the generation of reducing equivalents [NAD(P)H, FADH*2*].

### 3.3 Scale-up validation: shake flasks to 30 L fermenter

**FIGURE 7:**
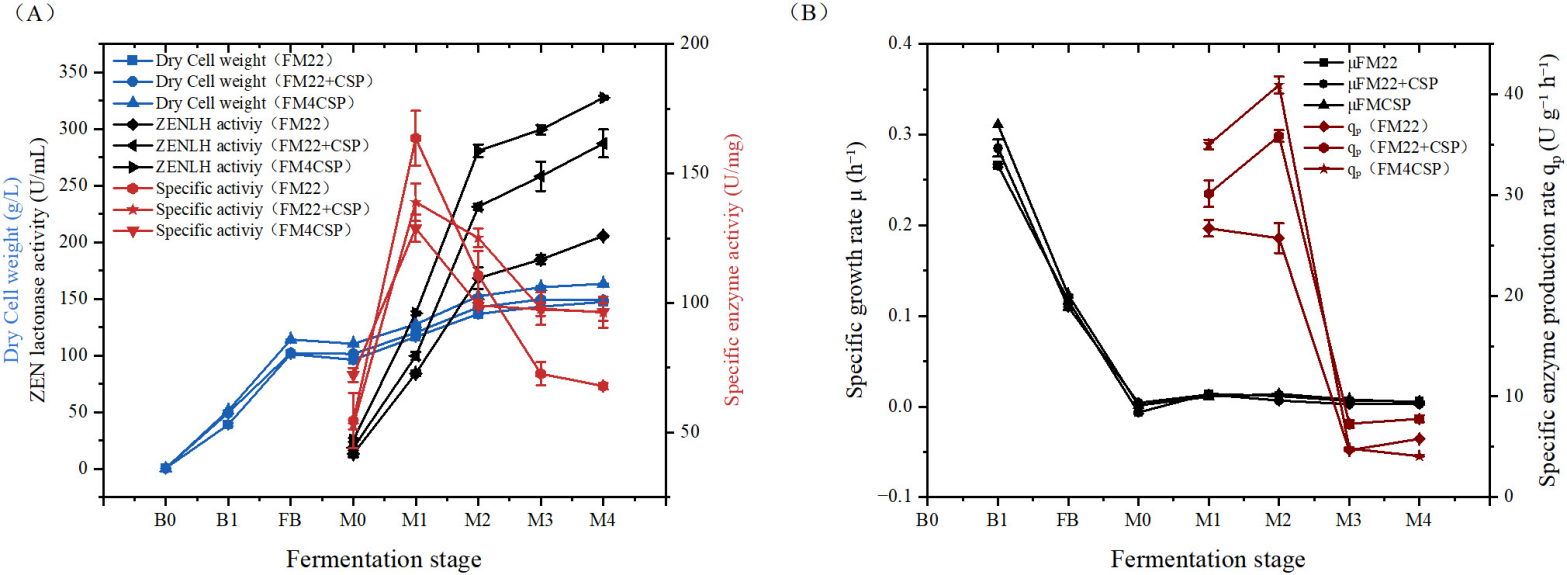
Scale-up fermentation of ZEN lactonase in a 30 L bioreactor: **(A)** cell growth and ZEN lactonase production profiles; **(B)** kinetics of specific growth rate and specific enzyme production rate. FM4CSP increased ZEN lactonase activity by 1.59-fold (327.56 U/mL) compared with conventional FM22 (205.52 U/mL), and CSP supplementation enhanced the specific production rate (*q*_p_) during the high-production phase.

To evaluate the transferability of FM4CSP medium from shake-flask systems to bioreactors, ZEN lactonase expression was assessed in a 30 L fermenter using three media: FM22, FM22 supplemented with CSP (19.18 g/L), and FM4CSP. As shown in Figure 7A, FM4CSP enhanced both cell growth and ZEN lactonase production. Biomass reached 163.30 ± 0.31 g/L in FM4CSP, compared with 147.24 ± 0.60 g/L and 148.87 ± 0.70 g/L in FM22 and FM22 + CSP, respectively. Analysis of the specific growth rate (Figure 7B) indicated that biomass accumulation occurred primarily during the batch and glycerol FB phases. This improvement may be attributed to the proliferative effect of CSP, together with reduced inorganic salt concentration and osmotic pressure in FM4CSP, which synergistically enhance cell growth.

In bioreactor systems, ZEN lactonase expression was achieved in FM22 medium without organic nitrogen supplementation, reaching an activity of 205.52 ± 1.06 U/mL. Supplementation of FM22 with CSP (19.18 g/L) ZEN lactonase activity increased to 287.16 ± 12.30 U/mL, representing a 1.40-fold increase compared with FM22. FM4CSP further enhanced ZEN lactonase expression, achieving an activity of 327.56 ± 1.78 U/mL, which is 1.59-fold higher than that observed in FM22 (Figure 7A). Analysis of specific enzyme activity (Figure 7A) revealed that CSP supplementation transiently reduced specific enzyme activity during the early induction phase, consistent with the trend observed in shake-flask cultures. However, during the later induction phase, CSP helped maintain stable specific enzyme activity. Moreover, during the M0–M1 and M1–M2 induction stages, the specific production rate (*q*_*p*_) was substantially higher with CSP-supplemented media. The maximum *q*_*p*_ values were 26.69 ± 0.83 U⋅g^–1^⋅h^–1^ in FM22, 35.86 ± 0.59 in FM22 + CSP, and 40.91 ± 0.83 in FM4CSP (Figure 7B). These findings indicate that CSP supplementation significantly enhances *q*_*p*_ during the high-production phase.

## 4 Discussion

Defined inorganic salt media (e.g., BSM, FM22) are considered ideal for the production of recombinant proteins in *Pichia pastoris* owing to their well-defined composition, batch-to-batch consistency, and downstream purification advantages (Matthews et al., 2018). However, they have long faced the challenge of low productivity during the methanol induction phase of methanol-utilizing fast (Mut^+^) strains when employed in shake-flask cultures (Žitkus et al., 2025). Consequently, most systematic optimization studies on such media at the shake-flask scale have used methanol-utilizing slow (Mut*^s^*/Mut^–^) strains as models, while those using Mut^+^ strains remain relatively scarce. To address these challenges, previous shake-flask-based studies either used complex media before switching to defined media in bioreactors or restricted such defined media to the glycerol phase. Moreover, bioreactor strategies were limited to simple dilutions or modifications to reduce precipitation and osmotic stress (Brady et al., 2001; Zhu et al., 2021). However, while these strategies enhanced production to a certain extent, none have entirely resolved the inconsistency between shake-flask and bioreactor media for Mut^+^ strains, and the underlying regulatory mechanisms remain unclear.

To bridge this knowledge gap, this study employed a DoE approach for systematically optimizing the bioreactor-derived FM224 medium for Mut^+^ strains. This effort resulted in the FM4CSP medium, a universal novel formulation that, with the same composition, supports high-level recombinant protein production in both shake-flask cultures and bioreactors. Our findings demonstrate that the choice of organic nitrogen source critically determines recombinant protein expression levels in shake-flask systems, with tryptone and CSP significantly outperforming yeast extract. Notably, although CSP supported lower specific activity, it achieved the highest volumetric activity. As a low-cost and readily available industrial byproduct rich in organic acids, amino acids, oligopeptides, and trace elements, CSP was selected as the optimal nitrogen source for further optimization (Zhou et al., 2022). These observations raise the following two key questions: (i) why is organic nitrogen indispensable in shake flasks, and why do different sources induce varying effects? (ii) What mechanisms enable organic nitrogen to bridge the performance gap between shake-flask and bioreactor systems?

Although microorganisms generally avoid prioritizing the synthesis of energetically expensive amino acids (Chen and Nielsen, 2022), previous studies have demonstrated that supplementing such amino acids in the culture medium can enhance heterologous protein expression (Heyland et al., 2011). Herein, under oxygen-limited shake-flask conditions, excessively rapid cell growth driven by free amino acids may suppress heterologous protein expression. Conversely, complex nitrogen sources provide not only readily available amino acids and peptides but also large peptides, which collectively support efficient enzyme production. Collectively, our results indicate that peptide molecular weight distribution appears to be the primary determinant of the effectiveness of organic nitrogen sources. Previous studies have indicated that nitrogen sources that require enzymatic hydrolysis for utilization (e.g., soya flour hydrolysate and peptone) act as competing substrates for proteases, thus reducing proteolytic cleavage of the target protein and enhancing heterologous protein production (Adivitiya et al., 2021). However, this protective mechanism does not explain the fundamental discrepancy between the strict dependence on organic nitrogen in shake-flask systems and its dispensability in fermenters.

To address this flask-to-fermenter discrepancy, a transcriptomic analysis was conducted to elucidate the underlying regulatory mechanisms. when *P. pastoris* (Mut^+^) uses methanol as the sole carbon source, methanol metabolism provides both carbon skeletons and energy to the cells (Jia et al., 2020; Bolmanis et al., 2022). This process is highly oxygen-dependent; therefore, oxygen limitation in shake-flask systems may impair methanol metabolism and consequently reduce ZEN lactonase expression (Gao et al., 2012). Consistent with this, ZEN lactonase activity in the optimized FM4 inorganic salt medium reached only 2.78 ± 0.15 U/mL (Supplementary Figure 1). Given that medium composition determines the extent of *de novo* amino acid synthesis (Heyland et al., 2011), supplementation with organic nitrogen sources reduces the need for endogenous synthesis and alleviates the metabolic burden. This shift improves cellular energy allocation and enhances ZEN lactonase production under shake-flask conditions. Organic nitrogen sources alleviate metabolic stress under oxygen-limited conditions by downregulating energy-intensive amino acid biosynthesis and reducing methanol metabolic burden.

Different sources of organic nitrogen trigger distinct metabolic regulatory patterns, which may be associated with variations in peptide composition. Specifically, yeast extract, which is primarily composed of small peptides (54.04% < 5 kDa, 78.95% < 8 kDa, Figure 4A) and free amino acids, can be directly assimilated as precursors for biosynthesis (Shang et al., 2017). This rapid nitrogen release generates a “nutrient-rich” signal that prioritizes cellular resource allocation toward growth (Figure 4C). Additionally, certain free amino acids may repress the methanol-inducible promoter (Rumyantsev et al., 2022), suppressing *AOX1* and enzyme induction. Consequently, the small-peptide composition of yeast extract leads to a growth-oriented metabolism with limited energy reallocation to recombinant protein production, explaining its modest effect on ZEN lactonase activity. In contrast, CSP is predominantly composed of large peptides (87.28% > 10 kDa, 100% > 8 kDa, Figure 4A) that function as slow-release nitrogen sources through extracellular hydrolysis (Hu et al., 2024). This gradual nitrogen supply enables cells to maintain metabolic homeostasis with minimal reprogramming, requiring only targeted downregulation of high-energy-cost AAA and lysine biosynthesis pathways to support efficient enzyme production. Thus, the high proportion of large peptides in CSP reduces the metabolic burden of de novo amino acid synthesis and preserves energy for heterologous protein production, resulting in higher ZEN lactonase activity. Tryptone, which contains a balanced proportion of small and large peptides (39.28% < 8 kDa, 60.72% > 8 kDa, 42.39% > 10 kDa, Figure 4A), provides both “immediate utilization” and a “sustained reserve.” This dual supply triggers systemic metabolic reprogramming. In addition to reducing energy demand for amino acid biosynthesis, tryptone downregulates pathways that generate reducing equivalents (*FDH*, *GND2*, *PDH*, *IDH*, and *SDH*), thereby preventing redox imbalance under oxygen-limited conditions. Concurrently, oxidative phosphorylation is upregulated to the highest level among the three nitrogen sources (Figures 5D,E; cluster K1). Such metabolic rewiring represents a canonical response to NADH-based reductive stress, in which cells reprogram their metabolism to restore redox homeostasis under oxygen-limited conditions (Yang et al., 2026). Thus, tryptone’s balanced peptides trigger downregulation of reducing-equivalent pathways and upregulation of oxidative phosphorylation, increasing ATP and alleviating redox imbalance to support high enzyme yields. Consistent with these observations, membrane fractionation experiments showed that the LMP derived from tryptone, despite containing only 40.84% of the total organic nitrogen, achieved enzyme activity comparable to that of intact tryptone (Figure 4C). These results provide direct evidence that large peptides are the primary functional components driving metabolic reprogramming. Moreover, upregulation of ABC transporters (cluster K5) may facilitate the uptake of exogenous peptides and amino acids from organic nitrogen sources, supporting efficient nitrogen assimilation under oxygen-limited conditions. Together, the limited oxygen supply in shake-flask systems constrains the efficient expression of ZEN lactonase in a minimal methanol medium. Supplementation with organic nitrogen sources alleviates this metabolic burden, thereby enabling high-level ZEN lactonase production in this system.

Under conventional shake-flask conditions (e.g., 250 mL flasks, 50 mL working volume, 200 rpm, without baffles), the maximum oxygen transfer rate (OTR_max_) is severely limited, typically to values well below 20 mmoL/L/h (based on data from Meier et al., 2016). In contrast, in stirred bioreactors equipped with active aeration, *P. pastoris* can achieve a high and stable oxygen uptake rate (OUR) of 200–300 mmoL/L/h during the methanol induction phase (Yu et al., 2010). Given the elevated oxygen transfer capacity of well-oxygenated bioreactors compared to shake flasks (Schulte et al., 2025), only an inorganic salt medium may support efficient expression. Consistent with this hypothesis, our bioreactor data indicated that FM22 supported substantial ZEN lactonase production when dissolved oxygen was adequately controlled, confirming that oxygen availability is the key factor distinguishing shake flasks from bioreactors in performance. The addition of CSP to FM22 further increased ZEN lactonase yield because CSP enhanced *q*_*p*_, indicating that the improvement in enzyme production was primarily due to an increase in per-cell productivity. Notably, even in bioreactors, when an oxygen-limited induction mode was applied, insufficient oxygen supply remained a limiting factor for heterologous protein expression, suggesting that CSP may also alleviate energy metabolic stress under such conditions. Therefore, organic nitrogen sources improve productivity in the two systems through a unified mechanism: metabolic relief is beneficial at any cultivation scale, although it becomes indispensable only under oxygen-limited conditions. Furthermore, the low-salt formulation of FM4CSP is suitable for shake flasks and bioreactors, further helping to bridge the gap between the two systems. Furthermore, CSP is a low-cost agricultural byproduct, and its successful validation at 30 L scale supports further scale-up to larger fermenters.

This study presents a novel universal medium (FM4CSP) that is applicable in both shake flasks and fermenters. It features a low-salt formulation, low-cost corn steep powder (CSP) as a key component, systematic DoE-guided optimization, transcriptomic mechanism elucidation, and multi-scale validation—features that distinguish it from previous empirical modifications of mineral salt media. Nevertheless, several issues remain to be addressed. For example, there is an interaction between biomass and nutrient components in the culture medium. High-cell-density fermentation is commonly employed in bioreactors (Duman-Özdamar and Binay, 2021), yet we applied the supplementation levels optimized for shake-flask conditions, which may be inappropriate. Furthermore, although we have elucidated the role of complex organic nitrogen sources such as CSP, their compositional complexity suggests that other components may also contribute to the effects observed. For example, transcriptomic data showed an upregulation of the fatty acid catabolic pathway. Consistent with this finding, oleic acid and alpha-linolenic acid further enhanced total enzyme and specific activities (Supplementary Figure 3). Beyond carbon-based strategies, such as methanol/sorbitol co-feeding (Jia et al., 2017a), these findings highlight the critical role of nitrogen sources in mitigating oxygen limitation. Thus, this work provides a complementary approach to bridging the shake-flask-to-bioreactor gap.

## 5 Conclusion

A novel medium (FM4CSP) compatible with both shake-flask and fermenter cultivation was developed using a combination of DoE and rational analysis. This formulation employs CSP as a low-cost, slow-release nitrogen source that alleviates the energy metabolic burden and maintains stable amino acid levels. Consequently, it effectively bridges the performance gap between shake-flask and fermenter systems and enhances heterologous protein expression in *P. pastoris*. Using the same FM4CSP medium, ZEN lactonase activity reached 25.87 ± 0.52 U/mL in shake flasks and 327.56 ± 1.59 U/mL in a 30 L fermenter, both of which are significantly higher than those achieved with the conventional FM22 medium.

## Data Availability

The datasets presented in this study can be found in online repositories. The names of the repository/repositories and accession number(s) can be found at: https://www.ncbi.nlm.nih.gov/, PRJNA1476329.

## Footnotes

1
www.biocloud.net
