# Supplementary material for: A universal medium bridging the shake-flask to fermenter gap in Pichia pastoris for enhanced zearalenone lactonase production
Source: Front Microbiol. 2026 Jul 1;17:1854154. doi: 10.3389/fmicb.2026.1854154 (PMC13368732; doi:10.3389/fmicb.2026.1854154)
Supplement: Supplementary file 1 [file Table_1.DOC]

**Supplementary material**

Table 1 Effects of different culture media on ZENLH production and pH value.

| Exp. S/N | Enrichment media | Induction media | ZENLH activity (U/mL) | pH value |
| --- | --- | --- | --- | --- |
| 1 | BMGY | FMM224 | 0 | 3.23–6.50 |
| 2 | BMGY | BMMY | 9.30 ± 0.19 | 6.28–6.50 |
|  | FMG224 | FMM224 | 0 | 3.52–6.50 |
| 4 | FMG224 | BMMY | 12.58 ± 1.00 | 6.21–6.50 |
| 5 | FMG224 | FMMPBS^a^ | 0 | 3.24–6.50 |
| 6 | FMG224 | FMMAD^b^ | 1.54 ± 0.06 | 6.21–6.50 |

^a^FMM224 medium with PBS buffer.

^b^FMM224 medium with periodic pH adjustment (6.5 via NaOH every 24 h).

Table 2 Plackett-Burman experimental design matrix for screening composition of the induction medium.

| Treatment | A | B | C | D | E | F | G | H | I | ZENLH activity(U/mL) |
| --- | --- | --- | --- | --- | --- | --- | --- | --- | --- | --- |
|  | Yeast extract | Tryptone | CaSO₄ | (NH₄)₂SO₄ | MgSO₄ | PTM5 | Biotin | KH₂PO₄ | K₂SO₄ |  |
| 1 | 0.00 | 2.00 | 0.10 | 6.25 | 3.63 | 0.33 | 0.125 | 13.40 | 4.50 | 8.35±0.49 |
| 2 | 1.00 | 0.00 | 0.25 | 6.25 | 3.63 | 0.33 | 0.1 | 10.70 | 4.50 | 1.86±0.03 |
| 3 | 0.00 | 2.00 | 0.25 | 6.25 | 2.90 | 0.33 | 0.1 | 13.40 | 3.60 | 3.89±0.49 |
| 4 | 1.00 | 2.00 | 0.10 | 6.25 | 3.63 | 0.50 | 0.1 | 10.70 | 3.60 | 6.86±0.39 |
| 5 | 1.00 | 0.00 | 0.10 | 5.00 | 3.63 | 0.33 | 0.125 | 13.40 | 3.60 | 1.68±0.06 |
| 6 | 0.00 | 0.00 | 0.10 | 5.00 | 2.90 | 0.33 | 0.1 | 10.70 | 3.60 | 1.64±0.03 |
| 7 | 1.00 | 2.00 | 0.25 | 5.00 | 2.90 | 0.33 | 0.125 | 10.70 | 4.50 | 2.59±0.07 |
| 8 | 0.00 | 2.00 | 0.25 | 5.00 | 3.63 | 0.50 | 0.125 | 10.70 | 3.60 | 8.02±0.82 |
| 9 | 0.00 | 0.00 | 0.10 | 6.25 | 2.90 | 0.50 | 0.125 | 10.70 | 4.50 | 2.13±0.19 |
| 10 | 0.00 | 0.00 | 0.25 | 5.00 | 3.63 | 0.50 | 0.1 | 13.40 | 4.50 | 7.28±0.03 |
| 11 | 1.00 | 2.00 | 0.10 | 5.00 | 2.90 | 0.50 | 0.1 | 13.40 | 4.50 | 6.73±0.05 |
| 12 | 1.00 | 0.00 | 0.25 | 6.25 | 2.90 | 0.50 | 0.125 | 13.40 | 3.60 | 6.46±0.72 |


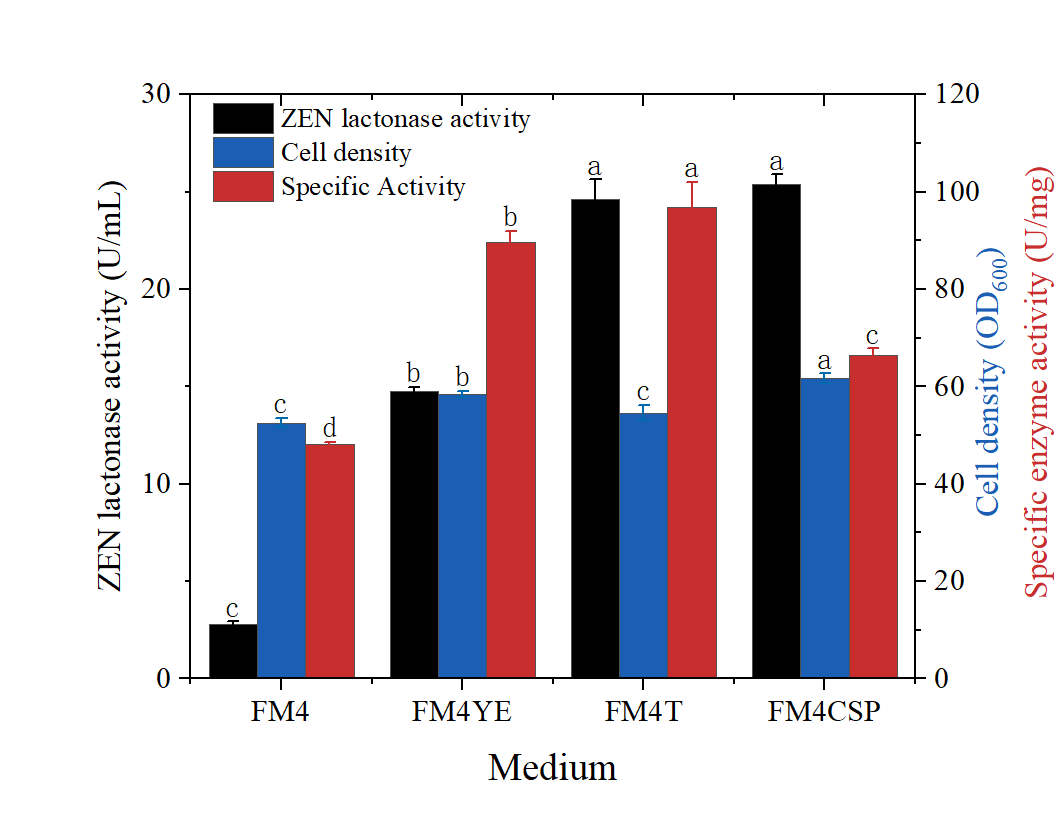


Figure. 1 Effects of Organic nitrogen sources on cell density and ZENLH production.

**
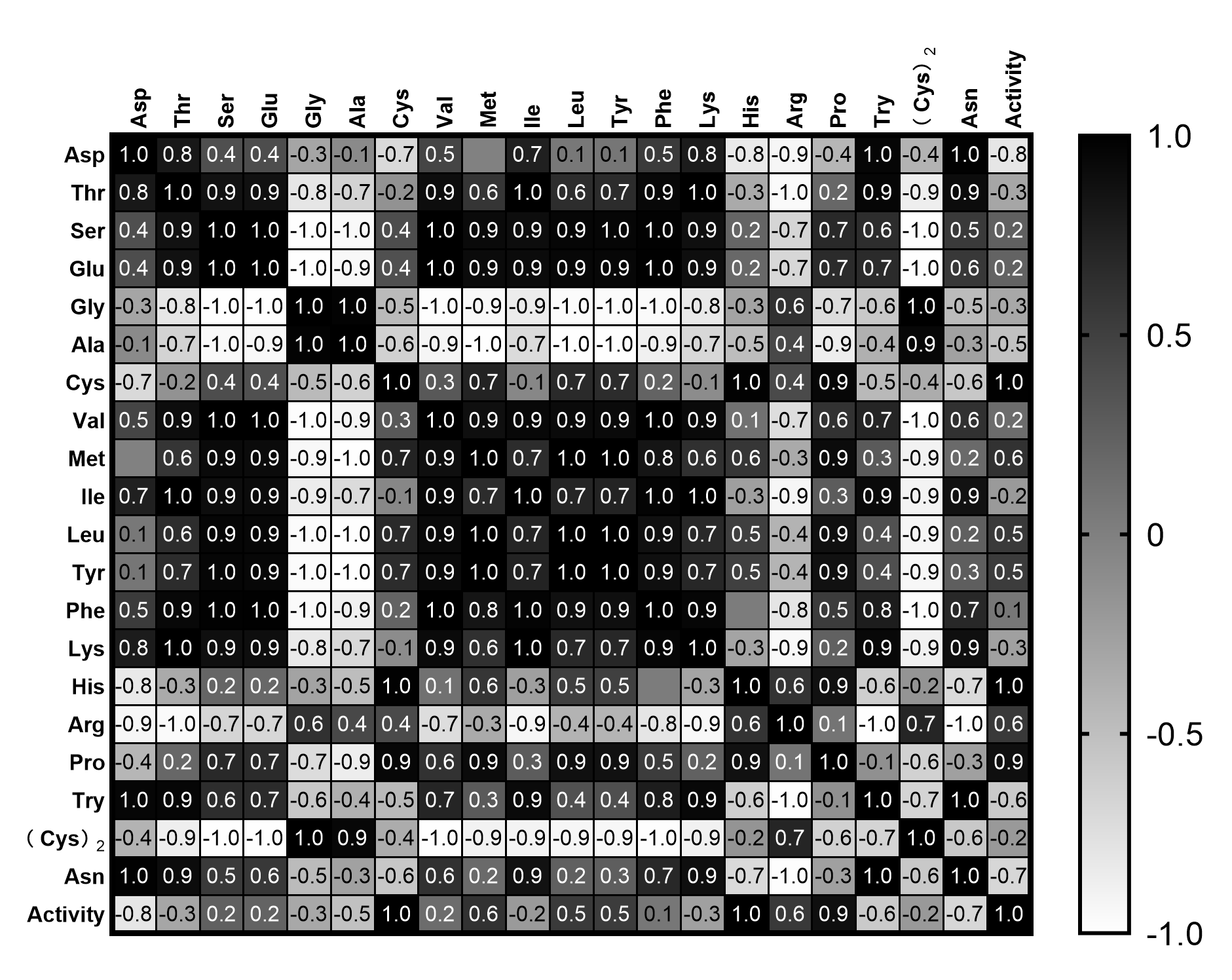
**

Figure. 2 Correlation analysis between ZENLH activity and amino acid Content (p<0.05).


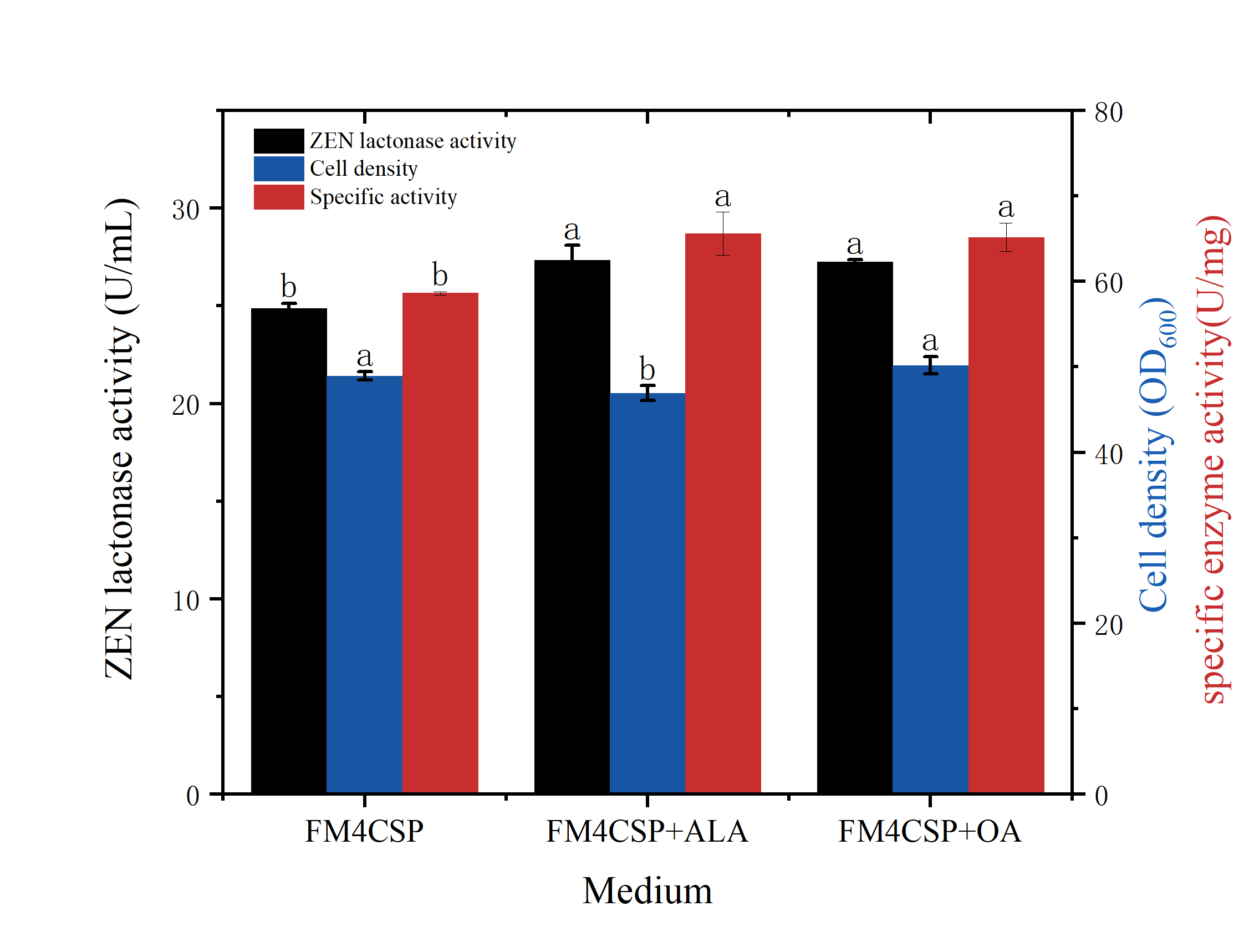


Figure. 3 Effects of oleic acid(OA) and alpha-linolenic acid (ALA) on cell density and ZENLH production.
